# Selective and Sensitive Detection of Cyanide Based on the Displacement Strategy Using a Water-Soluble Fluorescent Probe

**DOI:** 10.1155/2016/1462013

**Published:** 2016-01-05

**Authors:** Ming La, Yuanqiang Hao, Zhaoyang Wang, Guo-Cheng Han, Lingbo Qu

**Affiliations:** ^1^College of Chemistry and Molecular Engineering, Zhengzhou University, Zhengzhou 450000, China; ^2^College of Chemistry and Chemical Engineering, Pingdingshan University, Pingdingshan, Henan 467000, China; ^3^Henan Key Laboratory Cultivation Base of Nanobiological Analytical Chemistry, College of Chemistry and Chemical Engineering, Shangqiu Normal University, Shangqiu 476000, China; ^4^Changjun Middle School of Changsha, Changsha 410002, China; ^5^School of Life and Environmental Sciences, Guilin University of Electronic Technology, Guilin 541004, China

## Abstract

A water-soluble fluorescent probe (**C**-GGH) was used for the highly sensitive and selective detection of cyanide (CN^−^) in aqueous media based on the displacement strategy. Due to the presence of the recognition unit GGH (Gly-Gly-His), the probe** C**-GGH can coordinate with Cu^2+^ and consequently display ON-OFF type fluorescence response. Furthermore, the* in situ* formed nonfluorescent** C**-GGH-Cu^2+^ complex can act as an effective OFF-ON type fluorescent probe for sensing CN^−^ anion. Due to the strong binding affinity of CN^−^ to Cu^2+^, CN^−^ can extract Cu^2+^ from** C**-GGH-Cu^2+^ complex, leading to the release of** C**-GGH and the recovery of fluorescent emission of the system. The probe** C**-GGH-Cu^2+^ allowed detection of CN^−^ in aqueous solution with a LOD (limit of detection) of 0.017 *μ*mol/L which is much lower than the maximum contaminant level (1.9 *μ*mol/L) for CN^−^ in drinking water set by the WHO (World Health Organization). The probe also displayed excellent specificity for CN^−^ towards other anions, including F^−^, Cl^−^, Br^−^, I^−^, SCN^−^, PO_4_^3−^, N_3_^−^, NO_3_^−^, AcO^−^, SO_4_^2−^, and CO_3_^2−^.

## 1. Introduction

The development of effective methods for the recognition and sensing of anions has recently received considerable attention due to the importance of these species in biological and industrial processes [1–6]. Among various anions, cyanide (CN^−^) is considered to be one of the most toxic species due to its capability to bind strongly to ferric ions in cytochrome oxidase and reduce the activity of this enzyme [7]. Assimilation of CN^−^ through skin, lungs, and gastrointestinal tract can lead to convulsion, unconsciousness, and eventually death [8]. According to the World Health Organization (WHO), the maximum permissible level of CN^−^ in drinking water is 1.9 *μ*mol/L [9]. But, on the other hand, cyanide is extremely useful in various industrial processes such as gold mining, electroplating, metallurgy, and production of organic chemicals and polymers [10, 11]. The widespread industrial use of cyanide inevitably causes the accidental release of cyanide into the environment, therefore leading to serious problems. Consequently, it is highly required to exploit effective ways for monitoring the presence of cyanide anion [4, 12].

Various analytical methods and techniques have been developed for the determination of CN^−^, including titrimetry [13], voltammetry [14, 15], chromatography [16, 17], colorimetry [18, 19], and fluorometry [4, 12]. Many of these methods involve strict requirements of the sample handling, such as acidification of the CN^−^ followed by extraction of HCN. Therefore, quick methods that permit selective* in situ* determination of CN^−^ with high selectivity are highly required for medical point-of-care, industrial, and environmental online monitoring. Fluorometric assays based on optical probes possess innate advantages over other techniques, because of their simplicity of implementation, fast response times, high sensitivity, and excellent selectivity [20, 21]. Among the fluorescent probes, there are mainly four strategies for CN^−^ sensing: (i) coordination to electron-deficient center [15], (ii) nucleophilic addition to the electron-deficient *π*-system [22, 23], (iii) hydrogen-bonding interaction [24, 25], and (iv) metal-CN^−^ affinity (displacement approach) [26–29]. Among these four approaches, the displacement strategy, where CN^−^ extracts Cu^2+^ from the metal receptor complex to form stable Cu(CN)_*x*_, resulting in a detectable optical signal, has attracted special attention. Cu(II) complexes of fluorescent chromophores are usually nonfluorescent due to the paramagnetic quenching effect. Upon the addition of cyanide, CN^−^ ions react with copper ion in such nonfluorescent copper complex to form very stable Cu(CN)_*x*_ species. This results in the recovery of the fluorescence of the chromophore. Thus, a type of turn-on fluorescent CN^−^ probe has been developed based on Cu(II) ensembles. In designing such probe, fluorophores with high quantum yields are required in order to improve the signal-to-noise ratio. Additionally, a recognition unit with high affinity towards Cu^2+^ should be incorporated to ensure that fluorescence quenching occurs upon addition of Cu^2+^. In the literature, there are few reports on Cu^2+^-ensemble based receptors for the fluorescence turn-on optical detection of CN^−^. However, most of these receptors have the drawbacks of poor selectivity and/or poor water solubility.

In previous work, we reported a fluorescent probe** C**-GGH by incorporation of a natural tripeptide GGH (Gly-Gly-His) moiety to a coumarin fluorophore. Taking advantage of the natural tripeptide GGH, the probe exhibits excellent biocompatibility and water solubility [30]. Herein, we exploited this probe for the detection of CN^−^ based on a displacement sensing strategy. Due to the specific recognition of Cu^2+^ by GGH, the probe can bind with copper ions to form** C**-GHH-Cu^2+^ complex, and the paramagnetic Cu^2+^ center has a pronounced quenching effect on the fluorescence of the probe. Then, in the presence of CN^−^, Cu^2+^ can be released from the complex of** C**-GHH-Cu^2+^, resulting in the luminescence restoration of the probe. The ensemble** C**-GGH-Cu^2+^ allowed detection of CN^−^ in aqueous solution with high sensitivity and excellent selectivity.

## 2. Experimental

### 2.1. Materials and Instrumentation

All chemical reagents and solvents for synthesis were of analytical grade and commercially available and used as received without further purification unless otherwise stated. Deionized water (18 MΩ cm^−1^) from a water purification system (Simplicity Plus, Millipore Corp., Billerica, MA, USA) was used throughout.** C**-GGH (coumarin-Gly-Gly-His) was synthesized by following standard solid phase 9-fluorenylmethoxycarbonyl (Fmoc) chemistry according to our previously reported procedure [30].

The fluorescence spectra were carried out on a Varian Cary Eclipse spectrofluorimeter. The absorbance spectra were recorded on a Varian UV-Vis spectrophotometer. The excitation wavelength was 420 nm. The excitation and emission slit width were set both at 5 nm. All experiments were conducted at room temperature.

### 2.2. UV-Vis and Fluorescence Titration Experiments

The detection measurements were conducted as follows. First, the stock solution of the sensor was prepared by dissolving the sensor compound in HEPES buffered aqueous solution (10 mmol/L, pH 10.0) containing 1.0 mmol/L CTAB (cetyltrimethylammonium bromide). The stock solution of Na_2_S was prepared by dissolving Na_2_S in HEPES buffer (10 mmol/L, pH 10.0). Before the measurements, the probe stock solution and the CN^−^ stock solution were diluted with HEPES buffered water (pH 10.0), and then the diluted CN^−^ was added to the diluted probe solution; afterward, the fluorescence spectra were recorded immediately. All spectrum tests were carried out in a HEPES buffered (10 mmol/L, pH 10.0) aqueous solution containing 1 mmol/L CTAB.

### 2.3. Detection of CN^−^ in Real Sample

To begin with, 10 mL of cyanide-containing gold leach waste solution (taken from a gold mine tailings pond in Hunan, China) was diluted with 90 mL water. And the solution was filtered through a 0.22 *μ*m PTFE filter. Then 4 *μ*L of the above solution was added to 2 mL probe solution. Subsequently, various concentrations of CN^−^ were introduced into the above mixtures and the fluorescent spectra were recorded.

## 3. Results and Discussion

### 3.1. Absorption and Fluorescence Spectroscopy of ** C**-GGH to Cu^2+^

**Figure 1:**
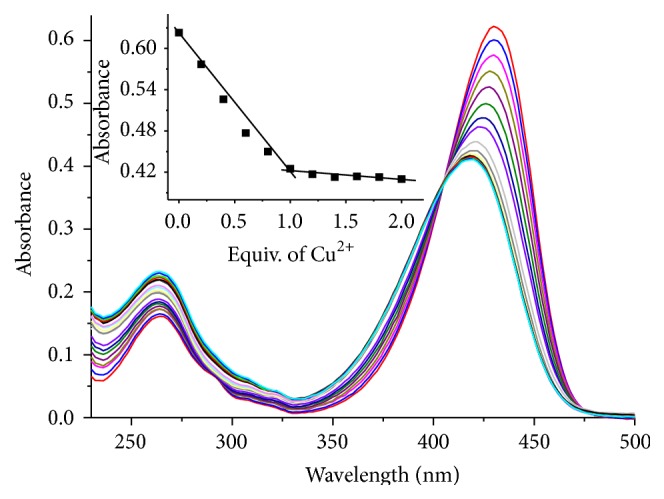
UV-Vis absorption responses of** C**-GGH (10 *μ*mol/L) upon the addition of different concentrations of Cu^2+^ (0–2 equiv.) in HEPES buffer aqueous solution (10 mmol/L, pH 10.0). Inset: the corresponding changes of absorption intensity of** C**-GGH at 430 nm in the presence of different concentrations of Cu^2+^.

The interaction of** C**-GGH with Cu^2+^ was investigated by UV-Vis spectrophotometric titration in HEPES buffer (10 mmol/L, pH 10.0). The UV-Vis absorption spectrum of** C**-GGH displayed a strong absorbance band centered at about 430 nm, which is the characteristic absorption profile of 7-diethylaminocoumarin-3-carboxylic acid. Titrations of Cu^2+^ with solutions of** C**-GGH led to substantial changes in UV-Vis spectra. As shown in Figure 1, upon an increase in the concentration of introduced Cu^2+^ (0-1 equiv. of Cu^2+^), the maximum absorbance intensity gradually decreased and shifted to about 410 nm, indicating the coordination of** C**-GGH to a paramagnetic Cu^2+^ center. Appearance of an isosbestic point at 405 nm also demonstrates the formation of a well-defined complex between** C**-GGH and Cu^2+^. The absorbance band was no longer changed noticeably when the concentration of Cu^2+^ increased from 1 to 2 equiv. (Figure 1, inset), suggesting 1 : 1 complexation of the probe with the Cu^2+^ ion.

**Figure 2:**
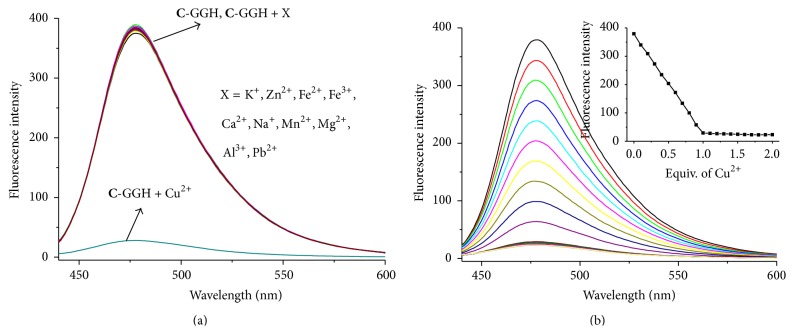
(a) Fluorescence responses of** C**-GGH (1 *μ*mol/L) to different ions (2 equiv. for K^+^, Zn^2+^, Fe^2+^, Fe^3+^, Ca^2+^, Na^+^, Mn^2+^, Mg^2+^, Al^3+^, and Pb^2+^; 1 equiv for Cu^2+^) in HEPES buffer aqueous solution (10 mmol/L, pH 10.0). Fluorescence titration of** C**-GGH (1 *μ*mol/L) with Cu^2+^ (0–2 equiv.) in HEPES aqueous buffer (10 mmol/L, pH 10.0). Inset shows fluorescence changes of** C**-GGH at 478 nm as a function of the Cu^2+^ concentration.

The fluorescence spectra of** C**-GGH were obtained by excitation of the fluorophore at 430 nm in HEPES buffer (10 mmol/L, pH 10.0), and a strong emission peak was observed at 478 nm. First, to gain insight into the sensing properties of** C**-GGH, the emission characteristics were examined in the presence of various metal species. The addition of 2 equiv. of K^+^, Zn^2+^, Fe^2+^, Fe^3+^, Ca^2+^, Cd^2+^, Na^+^, Li^+^, Ba^2+^, Mn^2+^, Mg^2+^, Al^3+^, Pb^2+^, and Ni^2+^ had no obvious effect on the fluorescence emission of** C**-GGH (Figure 2(a)). When 1 equiv. of Cu^2+^ was added to the probe solution, dramatic fluorescent quenching (quenching  efficiency  (*I*_0_ − *I*)/*I*_0_ × 100 = 94%) was observed, suggesting that probe** C**-GGH shows a specific response to Cu^2+^ which can be ascribed to the selective coordination of GGH to Cu^2+^ and the chelation-enhanced fluorescence quenching (CHEQ) effect of the paramagnetic Cu^2+^ center.

**Figure 3:**
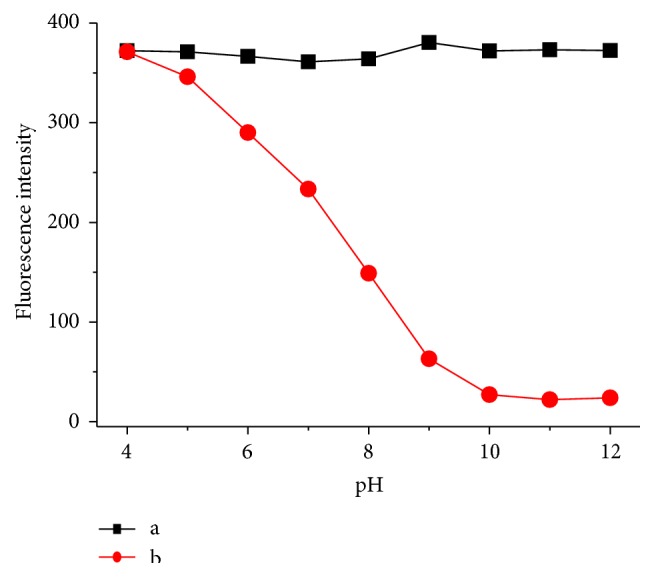
Fluorescence intensity of** C**-GGH at different pH: (a) free** C**-GGH, (b)** C**-GGH and Cu^2+^. Concentration of** C**-GGH is kept constant at 1.0 *μ*mol/L. Concentration of Cu^2+^ is 1.0 *μ*mol/L. Excitation is selected at 430 nm.

To quantitatively evaluate the luminescence response of** C**-GGH towards Cu^2+^, fluorescence titration with Cu^2+^ ions in varying concentrations was conducted. As shown in Figure 3(b), the emission band of** C**-GGH was gradually quenched upon addition of incremental amounts of Cu^2+^. The dose-dependent luminescence quenching shows a good correlation that can be expressed as *I* = 377.9 − 349.7 × ([Cu^2+^]/*μ*mol/L) (*R* = 0.999). The LOD (limit of detection) of** C**-GGH for determination of Cu^2+^ was calculated to be 21 nM (S/N = 3). This value is much lower than the maximum contaminant level (~20 *μ*mol/L) for copper in drinking water set by the U.S. Environmental Protection Agency [31], indicating that the probe** C**-GGH is sensitive enough to monitor Cu^2+^ for environmental assessment.

To investigate the range of pH in which** C**-GGH can effectively respond to Cu^2+^, we measured the titration curve of fluorescence intensity versus pH of the aqueous solution (Figure 3, curve a). The free** C**-GGH exhibited strong fluorescence in the studied pH range of 4–12. The effect of pH on the fluorescence of** C**-GGH-Cu^2+^ exhibited a quite different feature from that of the free probe** C**-GGH (Figure 3, curve b). The pronounced difference was observed in the range of pH > 10 in which the fluorescence intensity remained at a very low level. Clearly, the binding of Cu^2+^ caused this quenching. When pH was less than 10, the fluorescence intensity appeared to increase, and at pH 4, it reached the same level as that of the free probe** C**-GGH. This indicates that the** C**-GGH-Cu^2+^ complex dissociates into free ligand and ion, and thus Cu^2+^ causes no quenching to the fluorescence of** C**-GGH. The final pH value for this sensing system was set at 10.0.

### 3.2. Fluorescence Response of** C**-GGH-Cu^2+^ to CN^−^

**Figure 4:**
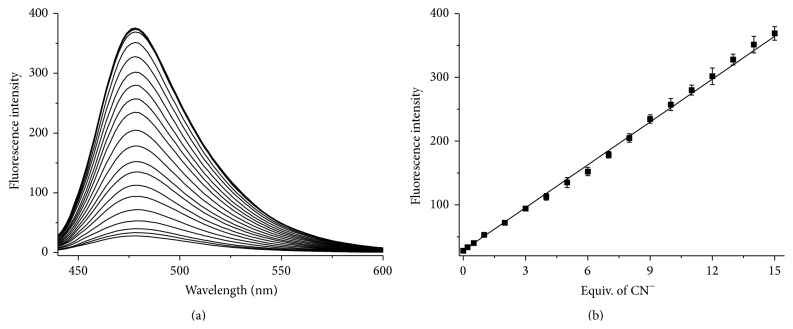
(a) Fluorescence titration of** C**-GGH-Cu^2+^ (1.0 *μ*mol/L) with CN^−^ (0–30 equiv., 0.0, 0.2, 0.5, 2.0, 3.0, 4.0, 5.0, 6.0, 7.0, 8.0, 9.0, 10.0, 11.0, 12.0, 13.0, 14.0, 15.0, 20.0, 25.0, and 30.0 *μ*mol/L, resp.) in HEPES aqueous buffer (10 mmol/L, pH 10.0). (b) Dose-dependent luminescence response of** C**-GGH-Cu^2+^ (1.0 *μ*mol/L) to CN^−^.

Cyanide anion can coordinate with Cu^2+^ to form a highly stable species Cu(CN)_*x*_ which has a much lower solubility product constant (*K* = 1.27 × 10^−24^) compared with that of** C-**GGH-Cu^2+^ (6.58 × 10^−6^) [30]. Therefore, the* in situ* formed** C-**GGH-Cu^2+^ complex could be a promising “OFF-ON” type luminescent sensor for the CN^−^ anion based on displacement approach. To investigate the luminescence response of** C-**GGH-Cu^2+^ towards CN^−^ in 100% aqueous media, a luminescence titration experiment was conducted by introducing different concentrations of CN^−^ into** C-**GGH-Cu^2+^ (1 *μ*mol/L) in 10 mmol/L HEPES buffer of pH 10.0. As shown in Figure 4, the emission intensity of the probe solution is increased steadily with incremental amounts of CN^−^, and on addition of about 15 equiv. of CN^−^ anion both the intensity and shape of the emission spectrum of** C-**GGH were completely restored. The dose-dependent luminescence enhancement shows good linearity over a concentration range of 0.2–15.0 *μ*mol/L, which can be expressed as *I* = 28.36 + 22.45 × ([CN^−^]/*μ*mol/L) (*R* = 0.998) (Figure 4(b)). The LOD of** C-**GGH probe for CN^−^ was calculated to be 0.017 *μ*mol/L which lies well below the limit of 1.9 *μ*mol/L for CN^−^ in drinking water set by WHO, indicating that the probe is sensitive enough for practical applications.

**Figure 5:**
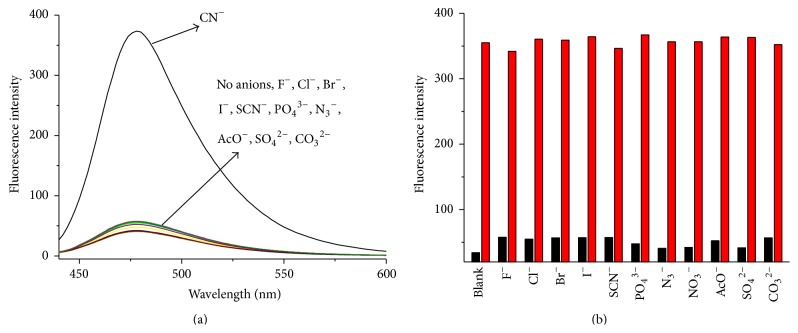
(a) Fluorescence changes of** C**-GGH-Cu^2+^ (1.0 *μ*mol/L) in the presence of various anions (15.0 *μ*mol/L) in HEPES aqueous buffer (10 mmol/L, pH 10.0). (b) Fluorescence responses of** C**-GGH (10 *μ*mol/L) at 478 nm in the presence of different anions (15.0 *μ*mol/L) (low bars), followed by addition of CN^−^ (15.0 *μ*mol/L) (high bars).

It is well known that the most significant behavior of a chemosensor is the high selectivity towards the analyte. In order to evaluate the selectivity of the proposed sensor for CN^−^, fluorescence responses of** C-**GGH-Cu^2+^ in the presence of various anions including F^−^, Cl^−^, Br^−^, I^−^, SCN^−^, PO_4_^3−^, N_3_^−^, NO_3_^−^, AcO^−^, SO_4_^2−^, and CO_3_^2−^ were measured. All these physiological and environmental important anions induced negligible fluorescence intensity changes (Figure 5(a)). Thus, the* in situ* generated** C-**GGH-Cu^2+^ complex can behave as a high selective luminescent “OFF-ON” sensor for CN^−^. To explore whether the** C-**GGH-Cu^2+^ complex could maintain its sensing response to CN^−^ in the presence of various other relevant interferences, competition experiments of** C-**GGH-Cu^2+^ were conducted.  Figure 5(b) shows the luminescence responses of** C-**GGH-Cu^2+^ to CN^−^ in the presence of various other anions. The results indicated that none of the other anions interfered with CN^−^ detection. All other anions only caused very weak background signal, while upon consequent addition of CN^−^ to each mixture, immediate enhancement in luminescence response was achieved.

### 3.3. Detection of CN^−^ in Real Sample

To assess the ability of** C-**GGH-Cu^2+^ for practical application, the probe was applied to monitor CN^−^ in a cyanide-containing gold leach waste solution. The accuracy of the assay was evaluated by spiking a known amount of standard CN^−^ solution and calculating its recovery. The results were summarized in Table 1. The recoveries of different known amounts of CN^−^ added were obtained from 96.0% to 102.0% with satisfactory analytical precision (RSD ≤ 4.3%), which confirmed the feasibility and reliability of the present probe.

## 4. Conclusions

In summary, a water-soluble fluorescent probe** C**-GGH was successfully used for recognition of Cu^2+^ and CN^−^ based on the displacement strategy. The probe** C**-GGH displayed high selectivity and sensitivity for copper ions in 100% aqueous solution. The* in situ* formed** C**-GGH-Cu^2+^ can effectively respond to CN^−^ accompanied by the fluorescence recovery of the probe system. The probe** C**-GGH-Cu^2+^ allowed detection of CN^−^ in aqueous solution with a LOD of 0.017 *μ*mol/L which is much lower than the maximum contaminant level (1.9 *μ*mol/L) for cyanide in drinking water set by the WHO. And the probe** C**-GGH-Cu^2+^ exhibited high selectivity for CN^−^ over other common anions. The proposed method was also successfully applied to detect the contents of CN^−^ in a cyanide-containing gold leach waste solution, which implies its great potential for the practical applications.

## Figures and Tables

**Table 1:** Analytical results of CN^−^ in a cyanide-containing gold leach waste solution.

Added CN^−^ (*μ*mol/L)	Found (*μ*mol/L)	Recovery (%)	RSD (*n* = 3) (%)
0	3.5		2.1
5.0	8.3	96.0	3.8
10.0	13.7	102.0	4.3
15.0	18.7	101.3	2.5
